# Enhanced Tolerance of Transgenic Potato Plants Over-Expressing Non-specific Lipid Transfer Protein-1 (*StnsLTP1*) against Multiple Abiotic Stresses

**DOI:** 10.3389/fpls.2016.01228

**Published:** 2016-08-22

**Authors:** Baniekal H. Gangadhar, Kappachery Sajeesh, Jelli Venkatesh, Venkidasamy Baskar, Kumar Abhinandan, Jae W. Yu, Ram Prasad, Raghvendra K. Mishra

**Affiliations:** ^1^Department of Molecular Biotechnology, Konkuk UniversitySeoul, South Korea; ^2^School of Applied Biosciences, Kyungpook National UniversityDaegu. South Korea; ^3^Department of Biological Sciences, University of Calgary, CalgaryAB, Canada; ^4^Amity Institute of Microbial Technology, Amity UniversityNoida, India; ^5^Amity Institute of Biotechnology, Amity UniversityGwalior, India

**Keywords:** abiotic stress-tolerance, drought, heat stress, non-specific lipid transfer protein, salinity, *Solanum tuberosum*

## Abstract

Abiotic stresses such as heat, drought, and salinity are major environmental constraints that limit potato (*Solanum tuberosum* L.) production worldwide. Previously, we found a potential thermo-tolerance gene, named *StnsLTP1* from potato using yeast functional screening. Here, we report the functional characterization of *StnsLTP1* and its role in multiple abiotic stresses in potato plants. Computational analysis of StnsLTP1 with other plant LTPs showed eight conserved cysteine residues, and four α-helices stabilized by four disulfide bridges. Expression analysis of *StnsLTP1* gene showed differential expression under heat, water-deficit and salt stresses. Transgenic potato lines over-expressing *StnsLTP1* gene displayed enhanced cell membrane integrity under stress conditions, as indicated by reduced membrane lipid per-oxidation, and hydrogen peroxide content relative to untransformed (UT) control plants. In addition, transgenic lines over-expressing *StLTP1* also exhibited increased antioxidant enzyme activity with enhanced accumulation of ascorbates, and up-regulation of stress-related genes including *StAPX, StCAT, StSOD, StHsfA3, StHSP70*, and *StsHSP20* compared with the UT plants. These results suggests that *StnsLTP1* transgenic plants acquired improved tolerance to multiple abiotic stresses through enhanced activation of antioxidative defense mechanisms via cyclic scavenging of reactive oxygen species and regulated expression of stress-related genes.

## Introduction

Plants growing in natural habitats are continuously subjected to multiple abiotic stresses such as drought, salinity, heat, cold and heavy metal stress. Adaptation and response to these stresses is extremely intricate and involve changes at molecular, cellular, and physiological levels. Among abiotic stresses, factors such as heat, drought, and salinity have a major impact on cultivated potato (*Solanum tuberosum* L.), affecting yield, tuber quality, and market value (Levy and Veilleux, 2007). In order to counter the negative effects of these abiotic stresses, scientists around the globe are engaged in developing broad-spectrum abiotic stress tolerant potatoes, but efforts have met with varying degree of success due to limited understanding of molecular mechanisms involved in abiotic stress-tolerance (Levy and Veilleux, 2007; Kappachery et al., 2015). In this scenario, it is important to identify potential candidate genes or gene networks associated with broad-spectrum multiple abiotic stress-tolerance. In our earlier publications, we reported 95 potential candidate genes responsible for imparting thermo-tolerance in potato using yeast functional screening approach (Gangadhar et al., 2014). Among 95 genes, 11 were found to be associated with multiple stress-tolerance (drought, salt and heat stress) in potato. The functional relevance of previously identified genes, *GLP1* (Germin-like protein 1), *nsLTP*1 (Non-specific lipid transfer protein1), *PI-PLC* (phosphoinositide-specific phospholipase-c), *CHP* (Conserved hypothetical protein) and *RPL4* (60 S Ribosomal L4/L1 protein) genes in improving abiotic stress-tolerance was confirmed by using virus induced gene silencing (VIGS) (Gangadhar et al., 2016). Herein, *StnsLTP1* is used as candidate gene for developing broad-spectrum multiple abiotic stress tolerant potato using genetic engineering.

Plant non-specific lipid transfer protein (nsLTPs) are low molecular mass basic proteins belong to the plant specific prolamin super family (Kader, 1997). To date a large number of LTPs have been described from various species, such as *Arabidopsis*, cotton, wheat, rice, and tobacco (Kinlaw et al., 1994; Feng et al., 2004; Liu et al., 2006; Boutrot et al., 2008). Besides mediating phospholipid transfer, plant LTPs have been implicated with the various biological functions, such as seed storage, lipid mobilization, cuticle synthesis, somatic embryogenesis and pollen tube adhesion (Carvalho and Gomes, 2007). LTPs have been found to modulate plant tolerance brought-out by environmental changes such as drought, cold, salt stress and also infection with bacterial and fungal pathogen (Sarowar et al., 2009; Choi et al., 2012; Guo et al., 2013). For instance, over-expression of pepper LTPs, *CALTP1* and *CALTP2* genes enhanced resistance to oomycete pathogen, *Phytophthora nicotianae* and bacterial pathogen, *Pseudomonas syringae* (Sarowar et al., 2009). More recently, over-expression of *Arabidopsis* nsLTP (*AtLTP3*) gene exhibited improved performance of *Arabidopsis* seedlings under salt, drought, and cold stress (Guo et al., 2013; Zou et al., 2013). However, the biological role of LTPs in improving abiotic stress-tolerance of potato is still an unsolved question. To date best evidence for the role of *StnsLTP1* in abiotic stress-tolerance came from our previous reports in which the heterologous expression of *StnsLTP1* in yeast cells (*Saccharomyces cerevisiae*) was found to enhance resistance to yeast cells to severe heat stress (Gangadhar et al., 2014). Later we confirmed its role in imparting improved thermo-tolerance in higher plants using VIGS (Gangadhar et al., 2016). The objectives of this study are to express a *StnsLTP1* gene in potato and to investigate its potentially increased resistance to multiple abiotic stress induced oxidative stresses by over-produced H_2_O_2_ via induction of antioxidant enzymes.

## Materials and Methods

### Plant Material and Growth Conditions

*In vitro* cultures of potato (*S. tuberosum*) cv. ‘Desiree’ were initiated from nodal segments and cultured in standard flat bottom culture boxes (100 mm × 110 mm) on Murashige and Skoog (MS) basal medium containing 2% (w/v) sucrose and gelled with 0.8% (w/v) plant agar (Duchefa, Germany). After every 4 weeks, nodal segments were excised and sub cultured on fresh MS medium for multiplication. The cultures were maintained at 24 ± 1°C with light: dark regime of 16: 8 h and light intensity of 40 μmol m^-2^ s^-1^ in growth chambers.

### Isolation and Gateway Cloning of *StnsLTP1* Gene

Full length cDNA encoding potato *StnsLTP1* (345 bp; Genbank accession- JX576237) was isolated from back transformed *Escherichia coli* plasmids containing specific cDNAs using gene specific prepared previously in our lab (Gangadhar et al., 2014; Supplementary Table S1). The coding region of *StnsLTP1*gene was prepared through PCR (95°C for 5 min, followed by 30 cycles of 95°C for 1 min, 55°C for 45 s and 72 °C for 5 min) using forward primer, 5- CACCATGGAAATGTTTGGCAAAATTGCAT-3 and the reverse primer, 5- TTACTGGAC CTTGGAGCAATCAGTG -3 containing CACC over hang at 5 end of the forward primer in order to clone into pENTR^TM^ /D-TOPO^®^ vector. The desired *StnsLTP1* DNA was PCR eluted according to manual instructions of FavorPrep GEL/PCR Purification Kit. Cloning of *StnsLTP1* was carried out using PCR amplification-based Gateway cloning method as described by Kumar et al. (2013). The purified *StnsLTP1* DNA fragments were cloned into pENTR^TM^ /D-TOPO^®^ vector using pENTR^TM^ Directional TOPO^®^ Cloning Kit (Invitrogen, Carlsbad, CA, USA), and the positive entry clones containing *StnsLTP1* gene was confirmed by colony PCR using M13 forward (-20) and M13 reverse primers (Supplementary Table S1). After confirming the sequence, entry clone including *StnsLTP1* gene was further mobilized into a plant expression vector PMDC32 using LR Clonase II^TM^ enzyme mix, (Invitrogen, Carlsbad, CA, USA), orientation and cloning of *StnsLTP1* gene was confirmed by colony PCR and DNA sequencing using PMDC32 specific forward and reverse primers as provided in Supplementary Table S1. The resulting construct, *StnsLTP1* gene under the control of the 2 × 35 S CaMV (cauliflower mosaic virus) promoter, were transferred into *Agrobacterium tumefaciens* GV3101 by using the gene pulser (Bio-Rad, Hercules, CA, USA).

### Development of *StnsLTP1* Over-Expression Potato Transgenic Lines

Transgenic potato plants expressing *StnsLTP1* gene were generated using *A. tumefaciens* mediated transformation according to the protocol described by Kappachery et al. (2015). Hygromycin resistant shoots were excised and transferred to the rooting medium (MS basal, 20 g l^-1^ sucrose, 250 mg l^-1^ cefotaxime, 25 mg l^-1^ hygromycin). Putative transformants were multiplied by nodal culture, and the insertion of the cassette carrying the transgene into genomic DNA was confirmed by PCR, Southern blotting and quantitative real-time reverse transcription PCR (qPCR). Genomic DNA was isolated from control and putative transgenic potato leaves according to Allen et al. (2006), and its purity was evaluated using Nano Drop ND-100 Spectrophotometer (Nano Drop Technologies, USA). Putative transgenic lines of potato were identified using hygromycin phosphotransferase (*hpt)* gene and pMDC32 vector specific primer flanking *StnsLTP1* gene (Supplementary Table S1). For Southern blotting, genomic DNA (30 μg) isolated from wild-type and putative transgenic plants were restrict digested with *EcoRI*, and separated on a 0.8% agarose gel. DNA was transferred onto a nylon membrane (Bio-Rad, USA) and hybridized with a probe for *hpt* gene. The probe was labeled using a Biotin DecaLabel kit (K0652, Fermentas, EU), and the membrane was then detected using the Biotin Chromogenic Detection Kit (K0661, Fermentas, EU), according to the manufacturer’s instructions.

To evaluate the possible role of the *StnsLTP1* gene in imparting tolerance to multiple abiotic stresses, we monitored expression levels of mRNA from different tissue of potato plantlets (leaf, stem, root, whole plant) subjected to heat, drought, and salinity stress treatments using qPCR. Total RNA was isolated from young leaves using Nucleo-Spin RNA plant mini prep kit (Macherey–Nagel, Germany) and genomic DNA contamination was removed with RNAse-free DNAse I (New England Biolabs, USA) treatment. First strand cDNA was prepared from 1 μg of total RNA using Accu-power rocket script RT premix kit (Bioneer, Korea). Real-time PCR amplification of the *StnsLTP1* gene was carried out using SYBR green master mix in Bio-Rad CFX96 real-time PCR system at 95°C for 10 min, followed by 29 cycles of 95°C for 20 s, 60°C for 45 s. Later, the products were analyzed through a melt curve analysis to check the specificity of PCR amplification. Each reaction was performed in triplicates, and the relative expression ratio was calculated using the 2^-ΔΔct^ method employing the *S. tuberosum actin* gene as the reference. The primers used in this study have been listed in (Supplementary Table S1).

### Stress Treatments

To assess the physiological and biochemical changes in plants under stress, 4-week old *StnsLTP1* transgenic and wild-type control plants grown in glasshouse conditions were subjected to different abiotic stress treatments. To assess thermo-tolerance, plants were initially acclimated by gradual increase in the temperature by 5°C in every 3 h, reaching to 30, 35, 40°C and finally maintained at 45°C for 24 h. For the salt stress, the plants were watered through trays underneath the pots with 200 mM NaCl solution for 15 days and drought stress was initiated by suspending watering the plants for 12 days. Non-stress treatment plants were maintained at 23 ± 1°C with regular irrigation with water (**Supplementary Figure S1**). All of the measurements of the physiological and biochemical parameters were performed on the fully expanded leaves. Cell membrane stability, cell viability, chlorophyll content, lipid per-oxidation [malondialdehyde (MDA) contents] and tissue-specific expression under different stress using qPCR was assessed from *StnsLTP1* transgenic and UT plants as described earlier (Gangadhar et al., 2016). Detailed protocol can be found in Supplementary Table S2.

### Quantification of Relevant Antioxidant Compounds

Total ascorbic acid was measured according to Davies and Masten (1991), as ascorbates acts as scavenger of toxic reactive oxygen species (ROS) including the superoxide radical ($O_{2}^{–}$), the hydroxyl radical (OH^-^) and hydrogen peroxide (H_2_O_2_). *StnsLTP1* transgenic and UT leaf samples (1 g) collected from stress and non-stress treatments was extracted using 1% of phosphate citrate buffer pH 3.5 using chilled mortar and pestle. The homogenate was then centrifuged at 10000 rpm at 4°C for 10 min. One milliliter of 2 mM 2,6- dichloroindophenol (2,6-DCPIP) was added to supernatant and the absorbance was recorded at 518 nm. The concentration of total ascorbic acid content was expressed as mg g^-1^ FW. For determination of reduced glutathione (GSH) content, the leaves of potato plants were homogenized in an ice bath with 5% (w/v) sulfosalicylic acid and centrifuged at 10000 rpm for 10 min. The supernatant was used for reduced glutathione (GSH) estimation and the GSH content was assayed following the change in absorbance at 412 nm after the addition of 5,5′-dithio-2,2′-dinitrobenzoic acid (DTNB) according to the method of Griffith (1980). The concentration of GSH content was expressed as μmol g^-1^ FW.

### Estimation of Hydrogen Peroxide Levels and Antioxidant Enzyme Activities

The hydrogen peroxide (H_2_O_2_) content in leaves collected from *StnsLTP1* transgenic and UT plants was determined by the ferrous ammonium sulfate/xylenol orange method as described by Cheeseman (2006). A standard curve was prepared from serial dilutions of H_2_O_2_ with concentration ranging from 100 to 1000 μmol ml^-1^ and *R*^2^ coefficient of 0.94. Data were normalized and expressed as micro-molar H_2_O_2_ g^-1^ FW of leaves.

To understand the signaling roles of *StnsLTP1* mediated up-regulation of antioxidant enzyme activities by providing tolerance to abiotic stresses induced oxidative damage was tested by measuring activities of antioxidant enzymes. Leaf samples (1 g) from the *StnsLTP1* over-expressed and UT potato plants subjected to different heat stress treatments as well as non-stress conditions were collected and homogenized using liquid N_2_. Protein was extracted using 0.1 M potassium phosphate extraction buffer (pH 7.5) containing 50% (v/v) glycerol, 16 mM MgSO_4_, 0.2 mM PMSF and 0.2% PVP at 4°C. The homogenate was centrifuged at 13,000 × g for 30 min at 4°C. Activities of ascorbate peroxidase (APX), catalase (CAT), superoxide dismutase (SOD) and Glutathione reductase (GR) were measured as described earlier (Hemavathi et al., 2011). The protein content of these enzyme extracts was determined using coomasive brilliant blue according to the method of Bradford (1976).

### Expression Profiling of Antioxidant and Stress-Responsive Genes Using Quantitative Real-Time PCR (qRT-PCR)

Total RNA isolation and cDNA synthesis was performed as described earlier from *StnsLTP1* transgenic and UT potato plants subjected to stress and non-stress conditions. All gene specific qPCR primers were designed based on their cDNA sequences are listed in Supplementary Table S3. The expression of genes encoding heat stress-responsive proteins and antioxidant enzymes such as *StHSP70* (encoding 70-kD heat shock protein), *StHSFA3* (encoding heat stress transcription factor A3), *StHSP90* (encoding 90-kD heat shock protein), *StsHSP20* (encoding class I small heat shock protein 20.1), *StAPX, StCAT, StSOD* and *StGR* was qPCR assayed using SYBR green qPCR kit as previously described (Gangadhar et al., 2014).

### Sequence Analysis of *StnsLTP1*

The pI (isoelectric point) and Mw (molecular weight) predictions were performed using the Compute pI/Mw tool^1^. The signal peptide was predicted using online SignalP, version 4.0^2^; Petersen et al., 2011). Multiple sequence alignments were carried out with ClustalX, version 1.83 (Thompson et al., 1997). Three dimensional structures (3D) of StnsLTP1 were generated using the SWISS-MODEL server (^3^Arnold et al., 2006). The details of sequences used for multiple sequence alignment analysis can be found in Supplementary Table S4.

### Statistical Analysis

All the experiments were independently carried out at least three times with transgenic and UT control lines, and each time with three replicates for all measurements. The means were analyzed using the Origin 8.1 program. The statistical differences between the treatment means were determined using one-way analysis of variance followed by Turkey’s test (*P* < 0.05), and standard error was calculated.

## Results

### Isolation and Sequence Analysis of *StnsLTP1* cDNA from Potato

**FIGURE 1:**
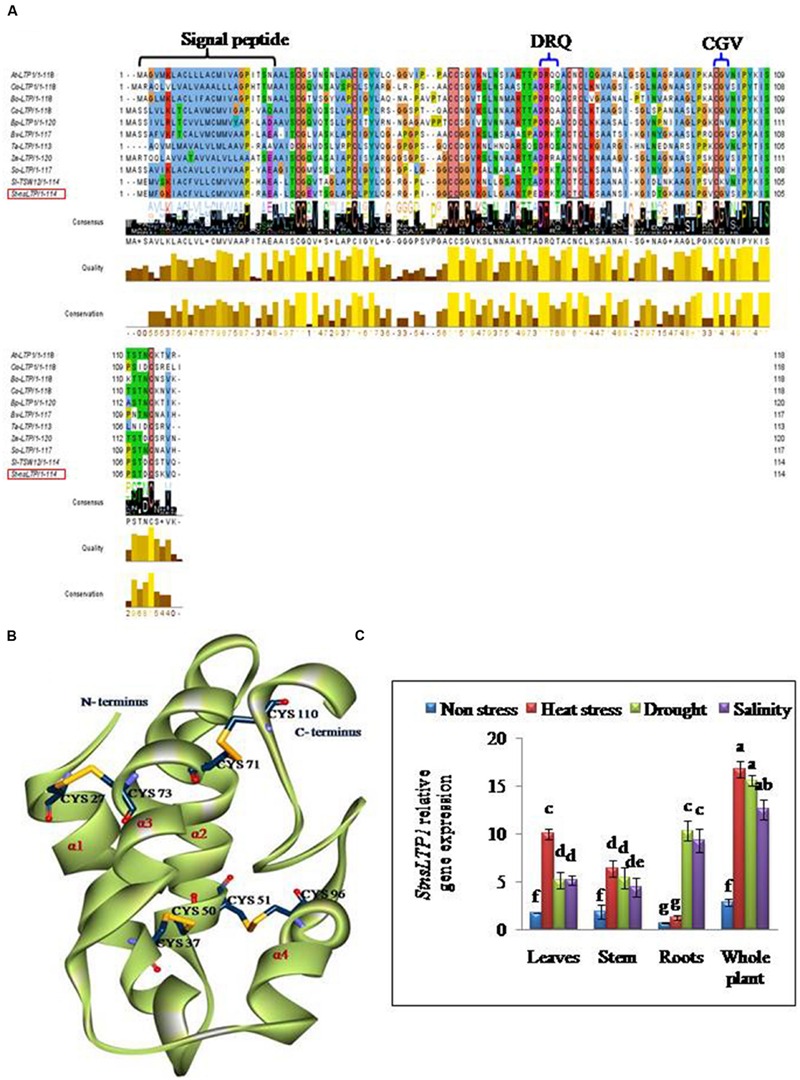
***StnsLTP1* sequence analysis and stress specific expression in wild-type potato plants. (A)** Alignment of the deduced StnsLTP1 sequence with other known plant nsLTP sequences. The amino acid sequences used in the alignment were from *Arabidopsis thaliana* (NP_181388.1), *Oryza sativa* (AAP92127), *Brassica oleracea* (AAA73948), *Castanea sativa* (ADK60918), *Betula platyphylla* (AFR31532), *Beta vulgaris* (Q43748), *Triticum aestivum* (P24296), *Spinacia oleracea* (AAA34032), *Solanum lycopersicum* (CAA39512), and *Zea mays* (P19656). The probable signal peptide sequence and lipid binding motifs (DRQ and CGV) in StnsLTP1 are indicated by flower bracket. The eight strictly conserved cysteine residues are boxed in black. **(B)** A 3D model of putative StnsLTP1 protein is shown as a ribbon model. The N-terminal and C-terminal are marked and the four α-helices are labeled α1–α4. The cysteine side chains forming the four disulfide bridges are showed as stick models. **(C)** Tissue-specific expression pattern of the *StnsLTP1* gene in control potato plants under heat, drought and salinity stress and non-stress using qRT-PCR.

Full length cDNA encoding *StnsLTP1* was isolated from potato cDNA expression library (Gangadhar et al., 2014) using gene specific primers. The deduced aminoacid sequence of *StnsLTP1*encodes an open reading frame containing 114 amino acids. The putative polypeptide of *StnsLTP1* contains a typical signal peptide of 24 amino acid residues at the N terminus and molecular mass of 8.7 kDa with theoretical pI of 8.8. Multiple amino acid sequence alignments showed that *StnsLTP1* contains the conserved lipid binding motifs such as DRQ and CGV and the eight conserved cysteine residues involved in formation of four disulfide bridges, following typical pattern of type I *nsLTP* {C X_2_ V X_6_ C [L] XY [L] X_12_ CC X G X_12_ DR [K] X_2_ CXC X_20_ P X_2_ C X_13_ C} as reported by Wang et al. (2012; **Figure 1**). Further sequence analysis with other plant *nsLTPs* revealed that *StnsLTP1* shares 83% sequence homology with *Solanum lycopersicum LTP* (*SlTSW12*; CAA39512) and more than 50 % homology with *Spinacia oleracea LTP* (*SoLTP*; AAA34032), *Beta vulgaris LTP* (*BvLTP*; Q 43748), *Betula platyphylla LTP* (*BPLTP*; AFR31532), which recently has been reported to be involved in imparting tolerance to several abiotic and biotic stresses in plants. The 3D structure of *StnsLTP1* was predicted using maize nsLTP (PDB Code: 1FK5) as a template, whose 3D structure has been determined by X-ray crystallography (Han et al., 2001). The hypothetical 3D structural model of *StnsLTP1* shows a protein that contains four α-helices, with a secondary structure compacted by four disulfide bridges and a C-terminal tail. The four bridges are formed by Cys27 and Cys73, Cys37 and Cys50, Cys51 and Cys26, and Cys71 and Cys110 (**Figure 1**). These results allow us to propose that this potato nsLTP (i.e., *StnsLTP1*) gene belongs to the type 1 nsLTP family.

### Tissue-Specific Expression Patterns of *StnsLTP1* under Various Abiotic Stresses

The results of qPCR analysis showed that the expression level of *StnsLTP1* was significantly different in different tissues under stress and non-stress conditions in UT potato plants (**Figure 1**). In absence of abiotic stress (heat, drought, and salt stress), *StnsLTP1* transcript accumulation was low in almost all organs tested, while its expression increased significantly upon heat stress in the leaves, stem and whole plant except roots. Whereas, upon drought or salt stress, expression of *StnsLTP1* was significantly higher in whole plant and roots compared other tissues tested. Thus, indicating that expression of *StnsLTP1* gene might responsible for inducing tolerance to multiple abiotic stresses.

### Molecular Analysis of Transgenic Potato Plants Over-Expressing *StnsLTP1*

**FIGURE 2:**
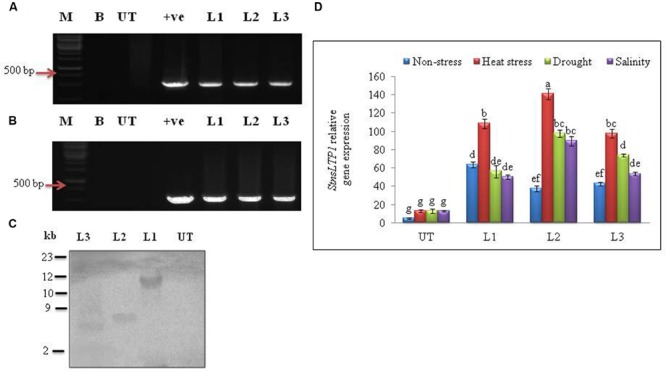
**Molecular analysis of putative transgenic potato lines over-expressing *StnsLTP1*. (A)** PCR amplification of *StnsLTP1* gene using gene specific primers. **(B)** PCR amplification using hygromycin phosphotransferase (*hpt*) gene. **(C)** Southern blot analysis of different independent potato control (Untransformed-UT), putative *StnsLTP1* transgenic lines (L1, L2, and L3). **(D)** The relative *StnsLTP1* expression levels in UT and transgenic lines (L1, L2, and L3) under non-stress as well as heat, drought and salinity stress conditions. M: 1000 bp DNA Ladder (+): positive control PCR from pMDC32 plasmid and B- water blank. Means of three independent samples and standard errors are presented. The same letters above the columns indicate no significant difference at *P* < 0.05.

*Agrobacterium*-mediated transformation of *StnsLTP1* gene and selection of hygromycin resistant transgenic plants produced 15 independent transformants. All of the 15 putative regenerated plants were PCR positive for both *StnsLTP1* (*StnsLTP1*gene flanked with pMDC32 vector) and *hpt* genes. Among them three representative transgenic lines (L1, L2, and L3) were selected for further studies (**Figures 2A,B**). Southern blot analysis of three transgenic lines showed successful integration of T-DNA into transgenic plant genomes, whereas no hybridization signal was detected in UT control plants. The variable size of gene on the blot indicated independent integration events in different transgenic plants (**Figure 2C**). The mRNA expression levels of *StnsLTP1* gene in putative transgenic lines is confirmed using qPCR. As shown **Figure 2D**, we noted that the expression of *StnsLTP1* in transgenic plants under different abiotic stresses (heat, drought, and salinity stress) was relatively higher in transgenic lines compared to UT control plants. Whereas, the levels of *StnsLTP1* expression was slightly increased in all three transgenic lines compared to UT control plants under normal growth conditions.

### Over-Expression of *StnsLTP1* Gene Enhances Tolerance to Heat, Drought, and Salt in Transgenic Potato

**FIGURE 3:**
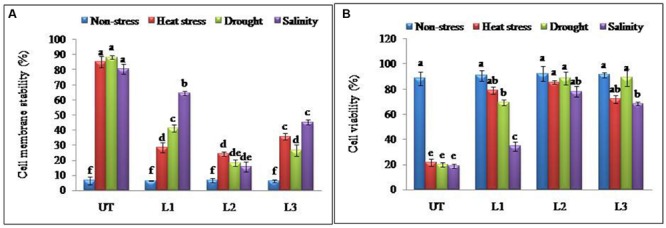
**Enhanced cellular adjustments in *StnsLTP1* transgenic lines **(A)** Percent membrane damage and **(B)** percent cell viability indicated by TTC reduction in *StnsLTP1* transgenic lines (L1, L2, and L3) and UT potato plants under, non-stress, heat, drought and salinity stress conditions.** Means of three independent samples and standard errors are presented. The same letter above the column indicates no significant difference at *P* < 0.05.

In the thermo-tolerance test, the *StnsLTP1* transgenic lines had higher survival rates than UT control plants. The survival rate of *StnsLTP1* transgenic plants (lines L1, L2, and L3) were 72, 89, and 70%, respectively, whereas UT control survival was only 10%. Drought induced by withholding watering produced similar results. In the salt tolerance test, *StnsLTP1* transgenic plants (lines L2 and L3) had survival rates of 77 and 54%, respectively, whereas the UT control plants had a survival rate of 7% (**Table 1**). These data suggest that *StnsLTP1* is a functional protein that improves the tolerance to multiple abiotic stresses in potato. To further confirm the multiple abiotic stress-tolerances observed in *StnsLTP1* over-expression lines, we carried out a quantitative assay for abiotic stress-tolerance based on cell membrane stability, cell viability and chlorophyll content of transgenic and UT plants subjected to stress and non-stress conditions (**Table 1**; **Figures 3A,B**). Upon abiotic stress, *StnsLTP1* over-expression lines (L2 and L3) showed notable improvements of cell viability, membrane stability index and reduced chlorophyll degradation than UT control plants. In summary, enhanced survival rates of *StnsLTP1* transgenic lines were associated with higher cell viability, membrane stability index and reduced chlorophyll degradation.

**Table 1:** Estimation of growth and biochemical parameters of *StnsLTP1* transgenic lines (L1, L2, and L3) and UT potato plants, during non-stress, heat, drought and salinity stress conditions.

Treatment	Shoot length (cm)	Root length (cm)	Survival rate (%)	Chlorophyll (mg g^-1^ FW)	Ascorbate content (mg g^-1^ FW)	GSH content (μmol g^-1^ FW)
**Non-stress**						
L1	14.0 ± 0.5b	9.1 ± 0.7a	96.4 ± 0.5a	9.2 ± 0.2ab	8.3 ± 0.1b	0.2 ± 0.01ab
L2	16.5 ± 0.1a	9.5 ± 0.8a	96.4 ± 0.7a	9.9 ± 0.2a	10.5 ± 0.8a	0.3 ± 0.02ab
L3	15.5 ± 0.5a	8.9 ± 0.3a	98.5 ± 0.6a	9.4 ± 0.1a	7.4 ± 0.7bc	0.2 ± 0.01a
UT	10.0 ± 0.5c	5.2 ± 0.8b	96.5 ± 0.3a	5.0 ± 0.4a	4.5 ± 0.3d	0.2 ± 0.01ab
**Heat stress**						
L1	11.0 ± 0.3c	6.7 ± 0.3c	72.6 ± 0.8b	5.2 ± 0.1c	14.3 ± 0.7b	1.0 ± 0.02a
L2	15.5 ± 0.9a	8.5 ± 0.3a	89.5 ± 0.4a	6.9 ± 0.6a	19.8 ± 0.2a	1.5 ± 0.03a
L3	13.5 ± 0.6b	7.5 ± 0.3b	70.6 ± 0.9bc	6.2 ± 0.9ab	18.9 ± 0.8a	0.8 ± 0.03ab
UT	2.0 ± 0.9d	1.0 ± 0.3c	10.7 ± 0.2d	2.2 ± 0.6d	5.2 ± 0.7c	0.3 ± 0.01c
**Drought**						
L1	7.5 ± 0.6b	4.5 ± 0.3b	66.4 ± 0.6b	4.4 ± 0.8c	10.6 ± 0.2c	0.9 ± 0.02ab
L2	11.0 ± 0.2a	7.3 ± 0.4a	78.4 ± 0.4a	7.3 ± 0.36ab	16.5 ± 0.9a	1.1 ± 0.01a
L3	11.0 ± 0.1a	7.5 ± 0.5a	76.4 ± 0.7a	7.6 ± 1.1a	14.7 ± 0.3ab	1.0 ± 0.01a
UT	2.1 ± 0.5c	0.8 ± 0.5c	12.5 ± 0.6c	1.7 ± 0.2d	4.9 ± 1.7d	0.2 ± 0.02c
**Salinity**						
L1	3.5 ± 0.8c	1.5 ± 0.3c	25.9 ± 0.9c	4.1 ± 1.1c	4.8 ± 0.3b	0.3 ± 0.01b
L2	10.5 ± 0.1a	6.3 ± 0.4a	77.1 ± 0.4a	5.7 ± 0.8a	12.7 ± 0.2a	1.2 ± 0.01a
L3	7.1 ± 0.2b	4.5 ± 0.5b	54.6 ± 0.2b	4.6 ± 0.4b	11.3 ± 0.3a	0.9 ± 0.02a
UT	2.0 ± 0.4d	0.8 ± 0.6d	7.7 ± 0.3d	1.7 ± 0.7d	4.7 ± 0.9b	0.3 ± 0.01b

*Means of three independent samples and standard errors are presented. The same letter indicates no significant difference at *P* < 0.05.*

### Over-Expression of *StnsLTP1* Gene Enhanced the Ability of ROS Scavenging under Multiple Abiotic Stresses

**FIGURE 4:**
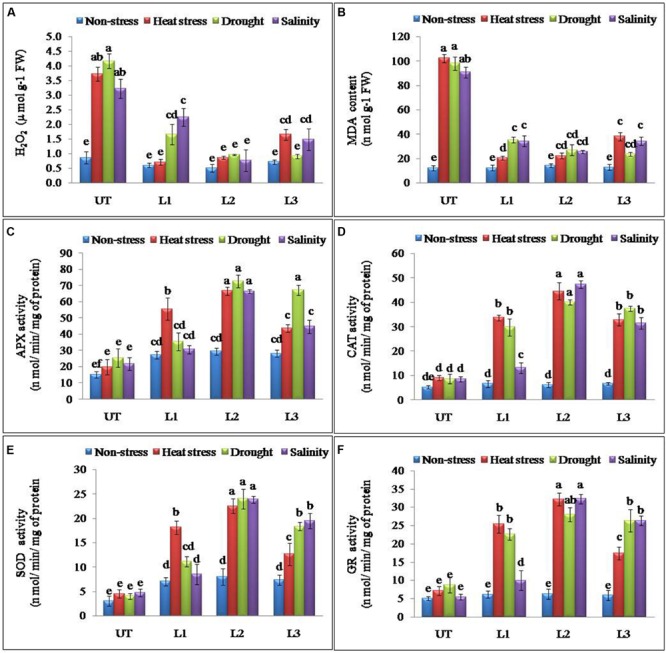
**Enhanced performance of *StnsLTP1* transgenic lines in scavenging ROS **(A)** H_2_O_2_ accumulation, **(B)** MDA contents and antioxidant enzyme activity of **(C)** APX, **(D)** CAT, **(E)** SOD and **(F)** GR in *StnsLTP1* transgenic lines (L1, L2, and L3) and untransformed (UT) potato plants under, non-stress, heat, drought and salinity stress conditions.** Means of three independent samples and standard errors are presented. The same letter above the column indicates no significant difference at *P* < 0.05.

Abiotic stresses result in the accumulation of ROS in plants. H_2_O_2_ is one of the most common ROS produced in the plants and its levels were found significantly higher in UT control plants as compared to the *StnsLTP1* transgenic potato plants under stress conditions (**Figure 4A**). *StnsLTP1* transgenic lines (L1 and L2) showed significantly reduced H_2_O_2_ production under heat and drought stress compared to UT control plants. In all the applied stress (heat, drought, and salinity stress) conditions as well as non-stress condition, UT control plants showed almost threefold higher levels of H_2_O_2_ as compared to the corresponding *StnsLTP1* transgenic plants. MDA, which causes membrane lipid per-oxidation, is a final product of the accumulation of ROS under stresses. Therefore MDA content in under stress can directly indicate the damage of the plants. The level of MDA as a consequence of heat, drought, and salinity stresses was increased in both *StnsLTP1* transgenic lines and UT plants. However, the transgenic lines accumulated less than three times (20–34 nmolg^-1^ FW) of MDA compared to UT control plants (90–102 nmolg^-1^ FW) under multiple abiotic stress conditions (**Figure 4B**). The specific activity of APX was increased 3.3, 2.8 and 3.0 times higher in *StnsLTP1* transgenic line (L2) compared to UT control plants when treated with heat, drought, and salinity stress respectively. Highest APX specific activity was recorded in *StnsLTP1* transgenic lines (L2 and L3) plants under heat stress while least activity was found in *StnsLTP1* transgenic lines (Ll) plants treated with 200 mM NaCl (**Figure 4C**). The specific activity of CAT was found 4.9 times higher in *StnsLTP1* transgenic line (L2) plants compared to UT control plants under all three abiotic stresses (**Figure 4D**). Highest SOD specific activity was recorded in *StnsLTP1* transgenic lines (L2) plants subjected to heat stress while least SOD activity was found in *StnsLTP1* transgenic lines (Ll) under salinity stress induced by 200 mM NaCl (**Figure 4E**). Similarly, specific enzyme activity of another major ROS scavenging enzyme, GR was measured and similar trends were observed (2.4–5.8 times increased activity) with highest GR activity recorded in *StnsLTP1* transgenic lines subjected to salinity stress induced by 200 mM NaCl, and least GR activity in control plants under salinity stress induced by 200 mM NaCl (**Figure 4F**).

### Over-Expression of *StnsLTP1* Gene Enhanced the Accumulation Antioxidant Compounds under Multiple Abiotic Stress Conditions

The ascorbate contents were measured in leaves of *StnsLTP1* transgenic lines (L1, L2, and L3) and the UT control plants grown under heat, drought, salinity and non-stress conditions (**Table 1**). Under heat, drought, and salt stress *StnsLTP1* transgenic line L2 registered 3.8, 3.3, and 2.7 fold higher accumulation of ascorbate compared to UT control plants, respectively. Similar to ascorbate, the reduced glutathione (GSH) contents in leaves of *StnsLTP1* transgenic lines grown under non-stress showed a slight increase over those of UT control plants (**Table 1**) and their levels dramatically increased with the application of heat, drought, and salinity stresses in *StnsLTP1* transgenic lines. This demonstrates that over-expression of *StnsLTP1* contributed to reduce the accumulation of ROS (H_2_O_2_) generated by stress mediated via enhanced accumulation of ascorbate and GSH in *StnsLTP1* transgenic lines.

### Over-Expression of *StnsLTP1* Gene Altered Expression of Antioxidant and Abiotic Stress-Responsive Genes

**FIGURE 5:**
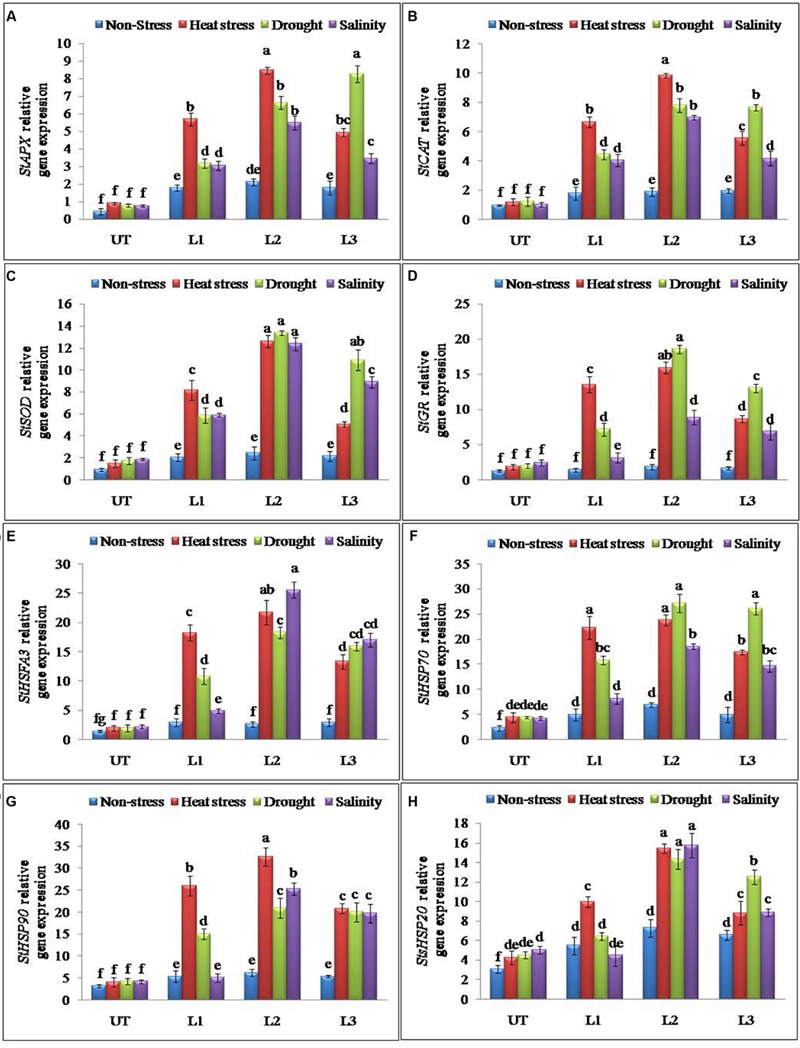
**Relative expression of antioxidant and genes encoding different stress-responsive proteins in *StnsLTP1* transgenic lines (L1, L2, and L3) and untransformed (UT) potato plants under, non-stress, heat, drought and salinity stress conditions. (A)** *StAPX*, **(B)** *StCAT*, **(C)** *StSOD*, **(D)** *StGR*, **(E)** *StHSFA3*, **(F)** *StHSP70*, **(G)** *StHSP90*, and **(H)** *StsHSP20.* Means of three independent samples and standard errors are presented. The same letter above the column indicates no significant difference at *P* < 0.05.

Antioxidant and ROS scavenging enzyme activities suggested that enhanced multiple abiotic stress-tolerance observed in *StnsLTP1* over-expression lines may involve in the selective up-regulation of antioxidant genes. To further support this observation, expression analysis of *StAPX, StCAT, StSOD* and *StGR* genes involved in ROS scavenging activities were studied under the multiple abiotic stress (heat, drought, and salt) treatments. Expression of genes *StAPX, StCAT, StSOD* and *StGR* were up-regulated in *StnsLTP1* transgenic lines compared to UT control plants under multiple abiotic stress (**Figures 5A–D**). Among different abiotic stress, *StSOD* and *StGR* genes showed maximum up-regulation under drought stress, whereas expression of *StAPX* and *StCAT* genes registered maximum up-regulation under heat stress (**Figures 5A–D**). Among the *StnsLTP1* transgenic lines, L2 showed about 9, 8, 7 and 9-fold higher expression of *StAPX, StCAT, StSOD* and *StGR* genes respectively, compared to their respective UT control plants under different abiotic stresses. Moreover, the mRNA expression levels of these antioxidant enzymes were positively correlated with their specific activities (**Figures 4C–F** and **5A–D**). To further investigate the molecular mechanism of stress-tolerance via *StnsLTP1* we analyzed the expression of down-stream genes (*StHsfA3, StHSP70, StHSP90* and *StsHSP20*) in transgenic potato. In the non-stress conditions, transcript levels of *StHsfA3, StHSP70, StHSP90* and *StsHSP20* were apparently enhanced in the three *StnsLTP1* transgenic lines compared with those of UT control. The expression of the *StHsfA3* gene improved concomitantly with stress treatments (except transgenic line L1 under NaCl) in transgenic lines compared to UT control plants (**Figure 5E**). About 11-fold expression (*StHsfA3*) was observed in L2 line under heat and salinity stress, while about ninefold expression was detected under drought stress compared to non-stress condition. Remaining L1 and L3 lines demonstrated relatively higher expression of *StHsfA3* gene under different abiotic stress conditions compared to UT control plants. Relative fold expression of *StHSP70* gene was found higher in *StnsLTP1* transgenic lines compared to UT plants under drought and heat stress treatments (**Figure 5F**). Compared to UT control plants, *StnsLTP1* transgenic line L2 showed maximum *StHSP70* gene expression about 6, 5, and 4-fold under drought, heat and salinity stress, respectively. However, *StHSP90* gene expression was up-regulated by sixfold in all three *StnsLTP1* transgenic lines under heat stress, while expression was increased by fourfold during drought and salinity stress (**Figure 5G**). Elevated *StsHSP20* gene expression about 2–3 fold was observed in *StnsLTP1* transgenic lines (except L1 under salinity and drought) compared to UT control plants under heat, drought and salinity stress conditions (**Figure 5H**). These data point out that over-expression of *StnsLTP1* in potato led to change in the transcript levels of endogenous stress-related and antioxidant genes.

## Discussion

Our previous study revealed that heterologous expression of *StnsLTP1* gene imparted enhanced tolerance to heat, drought, and salinity stress in wild-type yeast cells (Gangadhar et al., 2014). Further, we confirmed the functional role of *StnsLTP1* gene in imparting broad-spectrum thermo-tolerance to *Nicotiana benthamiana* using VIGS (Gangadhar et al., 2016). Herein, we demonstrated enhanced tolerance to multiple abiotic stresses (heat, drought, and salinity) through over-expression of *StnsLTP1* gene in potato for the first time. *StnsLTP1* encodes a small protein with eight highly conserved cysteine residues involved in formation of four disulphide bridges, as well as a 25-amino acid N-terminal signal peptide predicted to target proteins to the secretory pathway (Kader, 1997; Carvalho and Gomes, 2007). The compact and globular 3D structure of *StnsLTP1*, comprises of four α-helices and a flexible hydrophobic pocket with a distinctive ability to accommodate fatty acids, acyl-CoA, and other phospholipids (Kader, 1997; Carvalho and Gomes, 2007). The estimated molecular mass of mature *StnsLTP1 was* approximately 9 kDa, suggesting that this protein belongs to the type I LTP family (Kader, 1997; Carvalho and Gomes, 2007). In addition, *StnsLTP1* gene showed high similarity to the tomato LTP (*SlTSW12*), which is highly induced by various abiotic stresses such as NaCl, mannitol and ABA (Torres-Schumann et al., 1992). A homolog of *StnsLTP1* gene, *Betula platyphylla LTP* (*BPLTP*), which found to enhance the resistance of *E. coli* strain BL21 to salt (induced by NaCl) and drought (polyethylene glycol) stress upon its heterologus expression (Guan et al., 2013). Consistent with these results, *StnsLTP1* gene expression was found to be highly inducible in potato plants subjected to heat, drought, and salinity stress (**Figure 1C**). To gain further insight into the function of *StnsLTP1*, we generated transgenic potato plants over-expressing *StnsLTP1*. Over-expression of *StnsLTP1* in potato enhanced survivability during multiple abiotic stress and also suppressed the heat, drought, and salt-induced leaf damages by maintaining higher leaf chlorophyll content, antioxidant enzyme activity, and lower electrolyte leakage, H_2_O_2_ and MDA content (**Figures 3** and **4**; **Table 1**). Furthermore, cell death associated with cell structural damages, such as disruption of cell membranes and cell walls, which are typical heat and drought stress symptoms were high in UT control plants compared to *StnsLTP1* transgenic lines. These results indicated that over-expression of *StnsLTP1* facilitated better cellular survivability during multiple abiotic stress induced heat, drought, and salinity stresses and possibly be linked to the enhanced membrane integrity through reduced lipid per-oxidation, and accumulation of ROS such as H_2_O_2_ (**Figures 3** and **4**). In the present study, higher activities and transcripts of antioxidant enzymes *StAPX, StCAT, StSOD* and *StGR* were recorded in the transgenic lines (L1, L2, and L3) compared to UT control under normal growth conditions (**Figure 4**), suggesting that antioxidative defense system has been up-regulated by the over-expressed *StnsLTP1*. On the other hand, the expression of antioxidant enzymes (SOD, CAT, APX and GR) was increased by 5–9 folds in *StnsLTP1* transgenic lines subjected to multiple abiotic stresses as compared to UT control, suggesting that various components of ROS scavenging systems were co-regulated to induce the tolerance to multiple abiotic stresses in *StnsLTP1* transgenic lines (**Figure 4**). These results are consistent with our previous reports (Gangadhar et al., 2016), where silencing of *StnsLTP1* ortholog gene in *N. benthamiana* using VIGS produced hypertensive phenotype under heat stress with increased cell death and cell membrane damage. This finding demonstrates that *StnsLTP1* over-expression contribute to the reduce accumulation of ROS (H_2_O_2_) generated by stress through up-regulated expression of antioxidant enzyme genes, which in turn result in the elevated tolerance to heat, drought, and high salt-induced oxidative stresses. The negative effects of abiotic stress induced ROS accumulation is also counteracted by non-enzymatic low molecular weight metabolites such as ascorbate and glutathione. Furthermore, the enhanced accumulation of ascorbate and glutathione in potato is believed to play key role in modulating tolerance to various environmental stresses (Hemavathi et al., 2011). As a scavenger of ROS, both these metabolites (ascorbate and glutathione) accumulated in higher concentrations in *StnsLTP1* transgenic lines, which in turn helped plants to withstand the H_2_O_2_-mediated oxidative stress induced by different abiotic stress. Taken together, these observations suggests that over-expression of *StnsLTP1* gene resulted in decreased accumulation of ROS species (H_2_O_2_) mediated via increased accumulation of ascorbate and glutathione is proved to be an important factor for inducing multiple abiotic stress-tolerance. This is in agreement with the previous reports of Safi et al. (2015), over-expression of wheat LTP gene improved tolerance to oxidative stress induced by abiotic stress factors (100 mM NaCl, 50 mM JA and 20 mM ABA) through improved accumulation of ascorbates in *Arabidopsis*. Recently, the hypothesis that LTPs mediated expression of HSPs could be involved; in plant stress-tolerance induced by abiotic stress had emerged. It has been shown that over-expression of wheat LTP (*TaLTP3*) conferred thermo-tolerance through early accumulation of HSPs (AtHSP101and AtHSA32; Wang et al., 2014). Here, we tested *StnsLTP1* induced expression of HSPs such as *StHsfA3, StHSP70, StHSP90* and *StsHSP20* in transgenic lines over-expressed *StnsLTP1* subjected to different abiotic stress. Our results indicated that *StHsfA3* was up-regulated by 5-11-fold in *StnsLTP1* transgenic lines (L2 and L3) as compared to UT control (0.8–1 fold) under abiotic stress generated by heat, drought, or salinity stress. On the other hand, expression pattern of *StHSP70, StHSP90* and *StsHSP20* genes followed similar trend of expression with 3–4 fold higher expression in *StnsLTP1* transgenic lines compared to UT control potato plants. Based on these results, we suggest *StnsLTP1* mediated expression of *StHsfA3* may play important role in imparting tolerance to different abiotic stress in potato plants (**Figures 5E–H**). However, the molecular relationship between the putative protective roles of *StnsLTP1* induced expression of heat shock factors to various stress conditions needs to be determined.

In summary, *StnsLTP1* cloned from potato was classified as type I LTP and closely related to LTP genes in other Solanaceae species. Over-expression of *StnsLTP1* in potato alleviated cellular damages and growth inhibition due to multiple abiotic stresses such as heat, drought, and salinity stress. The improved tolerance to multiple abiotic stresses in *StnsLTP1* over-expressing lines of potato is manifested by greater survivability, chlorophyll content, and Cell membrane stability, as well as lower level of membrane lipid per-oxidation. Moreover, the multiple abiotic stress-tolerances exhibited in *StnsLTP1* transgenic lines is attributed to more robust activation of ROS scavenging system and coordinated activation of an array of stress-responsive genes at molecular level, leading to synthesis of a broad range of protective compounds such as ascorbate and glutathione. These results are warranted that *StnsLTP1* could be a candidate for improving the plant tolerance to environmental stress in other Solanaceae species using genetic engineering. Over-expression of *StnsLTP1* provides a novel strategy for endogenously adjusting H_2_O_2_ signaling during heat stress, induction of ROS scavenging system against cellular ROS accumulation for protection of crops from abiotic stress induced oxidative stress injury and future research into role of cross-talk among ROS and heat stress response signaling pathways in other crops could be a promising avenue.

## Author Contributions

BG, KS, JV, and VB performed wet lab experiments; BG, KS, JV, and JY performed computational analysis; BG, KS, JV, VB, KA, and JY. interpreted the results; BG, KS, JV, VB, KA, JY, RP, and RM wrote the manuscript.

## Conflict of Interest Statement

The authors declare that the research was conducted in the absence of any commercial or financial relationships that could be construed as a potential conflict of interest.

The reviewer AC and handling Editor declared their shared affiliation, and the handling Editor states that the process nevertheless met the standards of a fair and objective review.

## Footnotes

1 http://web.expasy.org/compute_pi/

2 http://www.cbs.dtu.dk/services/SignalP/

3 http://swissmodel.expasy.org/

## Supplementary Material

FIGURE S1 **Enhanced plant growth performances of *StnsLTP1* transgenic lines (L1, L2, and L3) and UT potato plants under, non-stress, heat, drought, and salinity stress conditions. (A)** Non-stress treatment plants were maintained at 23 ± 1°C with regular irrigation with water. **(B)** Heat stress was assessed by gradual increase in the temperature by 5°C in every 3 h, reaching to 30, 35, 40°C and finally maintained at 45°C for 24 h. **(C)** Drought stress was initiated by suspending watering the plants for 12 days. **(D)** Salt stress was assessed by irrigating the plants with 200 mM NaCl solution for 15 days. UT, untransformed plants; L1–L3, transgenic potato plants expressing *StnsLTP1* gene.
