# Supplementary material for: Enhanced Tolerance of Transgenic Potato Plants Over-Expressing Non-specific Lipid Transfer Protein-1 (StnsLTP1) against Multiple Abiotic Stresses
Source: Front Plant Sci. 2016 Aug 22;7:1228. doi: 10.3389/fpls.2016.01228 (PMC4993012; doi:10.3389/fpls.2016.01228)
Supplement: Supplementary file 1 [file Table_1.DOC]

**Supplemental Table S1. List of primers used in cloning and molecular characterization of *StnsLTP1* over-expression lines**

| **Name** | **Sequence (5̒ to 3̒)** | **Remarks** |
| --- | --- | --- |
| *StnsLTP1*-Forward | CACCATGGAAATGTTTGGCAAAATTGCAT | Isolation of full length StnsLTP1 gene from potato |
| *StnsLTP1*-Reverse | TTACTGGAC CTTGGAGCAATCAGTG |
| pMDC32 specific forward | TGTTTGAACGATCGGGGAAATTCGAGCTCC | Confirmation of insertion of gene into the expression caste and PCR validation of gene in *StnsLTP1* transgenic potato lines by PCR |
| pMDC32 specific reverse | GGATCCCCGGGTACCGG GCC |
| M13 forward (-20) | GTAAAACGACGGCCAG | Amplification of cassette carrying gene from pENTR™ Directional TOPO® vector |
| M13 reverse | CAGGAAACAGCTATGAC |
| *Hpt* forward | TTAGCGAGAGCCTGACCTATTGCATC | Identification of putative transgenic lines of potato by PCR |
| *Hpt* reverse | CAGAAGAAGATGTTGGCGACCTCGTA |
| *StnsLTP1*qRT- Forward | CCTGAAATCGGCAGCTAATTC | Expression profiling of StnsLTP1 gene in putative transgenic lines using qRT-PCR |
| *StnsLTP1*qRT- Reverse | GTGGAAGGGCTGATCTTGTA |

**Supplemental Table S2. Detailed protocol for determining cell membrane stability, cell viability, total chlorophyll contents and Lipid per- oxidation**

Cell membrane stability (CMS), cell viability (using TTC reduction assay) and chlorophyll content was assessed from *StnsLTP1* transgenic and NT plants as described earlier (Gangadhar et al., 2014b). Leaf discs subjected to abiotic and non-stress treatments were incubated in distilled water for 18 h at 25 ºC and electrolyte leakage (T1) was measured (**Hanna Instruments HI 2315 Digital, Bench-model Conductivity Meter)**. Total leakage (T2) was measured after autoclaving (15 min at 120 lbs) and CMS was calculated by [1 – *T1/T2*] X 100. For cell viability assay leaf discs were incubated in 0.4 % TTC solution (prepared in 50 mM sodium phosphate buffer pH 7.4) at 25 ºC under dark conditions. After 24 h of incubation, red color formazone was extracted by boiling with 5 ml of ethanol. To this another 5 ml of ethanol was added and absorbance was measured at 485 nm using a spectrophotometer (Shimadzu, ultraviolet 160A). The percent cell viability was calculated using the formula, [1 – T1/ T2] X 100; where T1- cell viability of stress sample and T2 – cell viability of non-stressed sample.

Chlorophyll content was determined by grinding 0.5 g leaf sample in 5 ml ice cold acetone and incubated overnight at 4 ºC. After incubation, the resultant mixture was centrifuged at 5000 × g for 5 min and supernatant thus obtained was made up to 25 ml volume using acetone and the absorbance of 1.2 ml aliquot was recorded at 663 and 645 nm using a spectrophotometer. The level of lipid per-oxidation in plant tissues was measured by determination of malondialdehyde (MDA) (Heath and Packer 1969) using thiobarbituric acid (TBA) reaction. About 1 g leaf tissue sample was homogenized in a solution of 0.5% (w/v) TBA in 20% (w/v) TCA. The homogenate was heated at 95°C for 30 min followed by cooling at ice and then samples were centrifuged at 12,000 rpm for 10 min. The absorbance of the resultant supernatant was measured at 532 and 600nm.The non-specific absorbance of the supernatant at 600 nm was subtracted from the maximum absorbance at 532 nm for MDA measurement. The level of lipid per-oxidation was expressed as nmol g^-1^ FW of MDA formed using an extinction coefficient of 155 mM-1 cm-1.

**Supplemental Table S3. List of real time PCR primers for antioxidant and stress responsive genes used in this study**

| **Genbank**  **accession** | **Name** | **Forward** | **Reverse** | **Length (bp)** | **Tm** |
| --- | --- | --- | --- | --- | --- |
| Z11982.1 | *StHSP70* | GGCATTGATCTTGGTACAACTTAT | GGAGTTGTTCTGTTGCCTTG | 95 | 61 |
| XM_006341106.1 | *StHSFA3* | CAGCTTTGTTCGACAGCTTAATAC | CAAATGCCTCTTCCCTCTCAA | 100 | 62 |
| AB041343 | *StAPX* | CTCCTCTGTGATCCTGCTTTC | GAGAGTGTCAAGTGAGCCTTAG | 98 | 62 |
| AY442179 | *StCAT* | CCATGCTGAGGTGTATCCTATTC | CCTTTCTCCTGGTTGCTTGA | 100 | 62 |
| AF354748 | *StSOD* | CATTGGAAGAGCTGTTGTTGTT | ATCCTTCCGCCAGCATTT | 96 | 62 |
| XM_006360298 | *StGR* | GCATTGCCGTTGCACTAAA | CTGAACGCATGGTCACAAAC | 102 | 62 |
| X55751 | *StActin* | GTTCCCTGGTATTGCTGATAGA | CTGTATTTCCTCTCTGGTGGAG | 99 | 62 |
| HS106768.1 | *StHSP90* | CAGTGGTATCAACGCAGAGTAA | TCCTTCACAGACTTGTCATTCTT | 107 | 62 |
| JX576239 | *StsHSP20* | GGAGAGAGGAATGTGGAGAAAG | CGCATTCTCCGGAAGTCTAAA | 102 | 62 |

**Supplemental Table S4. Details of plant LTP amino acid sequences used for multiple sequence alignment**

| **Id protein** | **Plant** | **Protein** |
| --- | --- | --- |
| NP_181388.1 | *Arabidopsis thaliana* | > At-LTP1  MAGVMKLACLLLACMIVAGPITSNAALSCGSVNSNLAACIGYVLQGGVIPPACCSGVKNLNSIAKTTPDRQQACNCIQGAARALGSGLNAGRAAGIPKACGVNIPYKISTSTNCKTVR |
| AAP92127 | Rice | >Os-LTP1  MARAQLVLVALVAAALLLAGPHTTMAAISCGQVNSAVSPCLSYARGLRPSAACCSGVRSLNSAASTTADRRTACNCLKNVAGSISGLNAGNAASIPSKCGVSIPYTISPSIDCSREL |
| AAA73948 | *Brassica oleracea* | >Bo-LTP  MAGLMKLACLIFACMIVAGPITSNAALSCGTVSGYVAPCIGYLAQNAPAVPTACCSGVTSLNNMARTTPDRQQACRCLVGAANALPTINVARAAGLPKACGVNIPYKISKTTNCNSVK |
| ADK60918 | *Castanea sativa* | >Cs-LTP  MASSLVLKLTCLAVMCMVIGAPVAQAAISCGQVQSSLVACIPYLRSGGSPTQACCNGVKSLNNAAKTTADRQAACECLKTAAGSISGLSPANAASLPGKCGVNVPYKISTSTNCKNVK |
| AFR31532.1 | *Betula platyphylla* | >Bp-LTP1  MASSIVLRLTCVVLMCMMVYAPLADAAVSCGQVQTSLLPCITYVRNNGAGAVPPTCCSGIVSVNNAAKTTPDRQAVCDCLKKAASALSGVNPNIIAGLPGKCNVNIPYKISASTNCKTIK |
| Q43748 | *Beta vulgaris* | >Bv-LTP  MASSAFVKFTCALVMCMMVAAPLAEAITCGLVASKLAPCIGYLQGAPGPSAACCGGIKSLNSAAASPADRKTACTCLKSAATSIKGINYGKAASLPRQCGVSVPYAISPNTNCNAIH |
| P24296 | *Triticum aestivum* | >Ta-LTP  AQVMLMAVALVLMLAAVPRAAVAIDCGHVDSLVRPCLSYVQGGPGPSGQCCDGVKNLHNQARSQSDRQSACNCLKGIARGIHNLNEDNARSIPPKCGVNLPYTISLNIDCSRV |
| P19656 | *Zea mays* | >Zm-LTP  MARTQQLAVVATAVVALVLLAAATSEAAISCGQVASAIAPCISYARGQGSGPSAGCCSGVRSLNNAARTTADRRAACNCLKNAAAGVSGLNAGNAASIPSKCGVSIPYTISTSTDCSRVN |
| AAA34032 | *Spinacia oleracea* | > So-LTP  MASSAVIKLACAVLLCIVVAAPYAEAGITCGMVSSKLAPCIGYLKGGPLGGGCCGGIKALNAAAATTPDRKTACNCLKSAANAIKGINYGKAAGLPGMCGVHIPYAISPSTNCNAVH |
| CAA39512 | *Solanum lycopersicum* | >Sl-TSW12  MEMVSKIACFVLLCMVVVAPHAEALTCGQVTAGLAPCLPYLQGRGPLGGCCGGVKNLLGSAKTTADRKTACTCLKSAANAIKGIDLNKAAGIPSVCKVNIPYKISPSTDCSTVQ |
| AFW90593.1 | *Solanum tuberosum* | >StnsLTP1  MEMFGKIACFVLLCMVVVAPRAEALSCGEVTSGLAPCLPYLQGRGPIGGCCGGVKGLLGAAKTPEDRKTACTCLKSAANSIKGIDTGKAAGLPGVCGVSIPYKISPSTDCSKVQ |
