# Supplementary figures and images for: Enhanced Tolerance of Transgenic Potato Plants Over-Expressing Non-specific Lipid Transfer Protein-1 (StnsLTP1) against Multiple Abiotic Stresses
Source: Front Plant Sci. 2016 Aug 22;7:1228. doi: 10.3389/fpls.2016.01228 (PMC4993012; doi:10.3389/fpls.2016.01228)

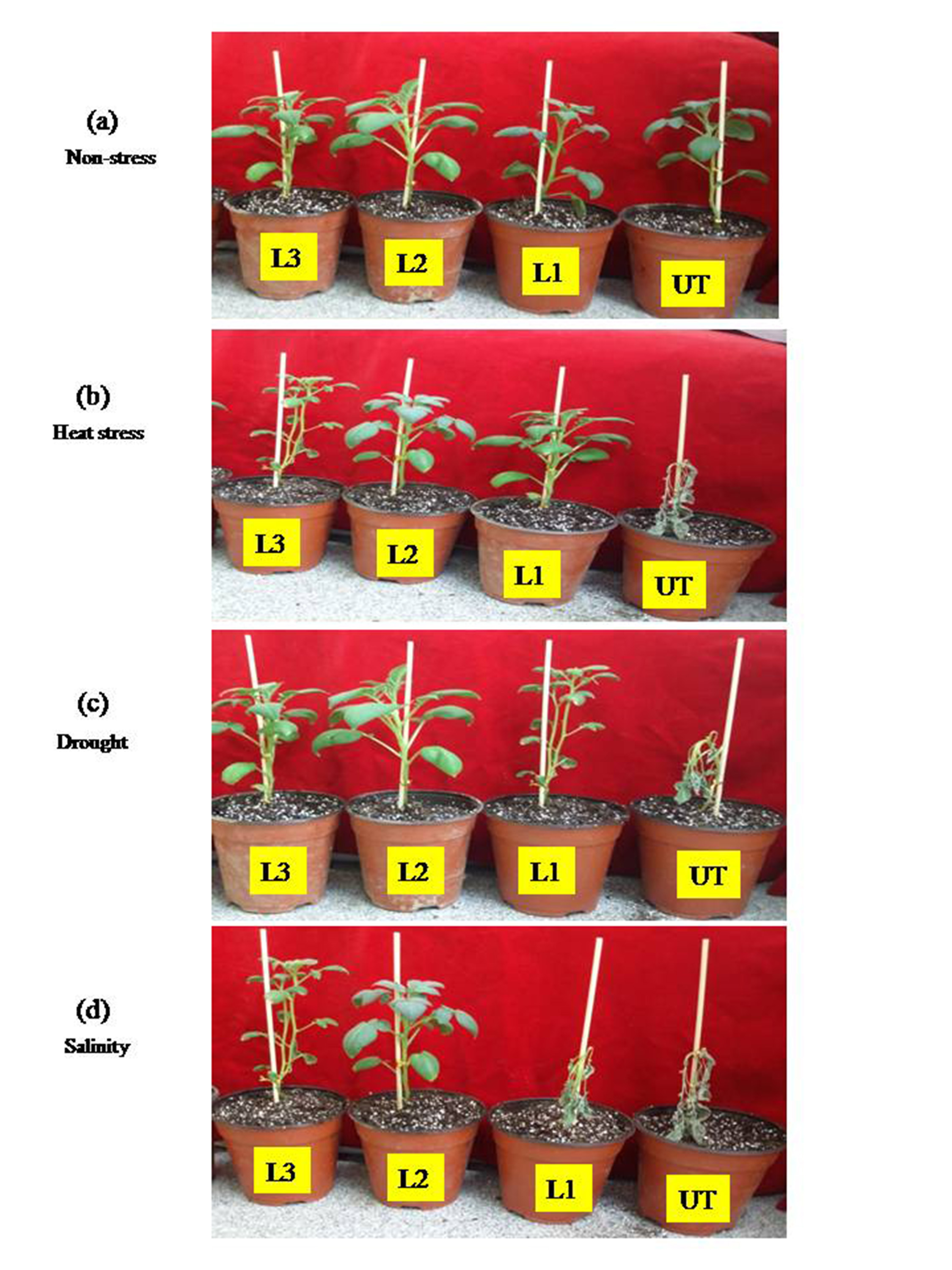

Supplement: FIGURE S1 — Enhanced plant growth performances of StnsLTP1 transgenic lines (L1, L2, and L3) and UT potato plants under, non-stress, heat, drought, and salinity stress conditions. (A) Non-stress treatment plants were maintained at 23 ± 1°C with regular irrigation with water. (B) Heat stress was assessed by gradual increase in the temperature by 5°C in every 3 h, reaching to 30, 35, 40°C and finally maintained at 45°C for 24 h. (C) Drought stress was initiated by suspending watering the plants for 12 days. (D) Salt stress was assessed by irrigating the plants with 200 mM NaCl solution for 15 days. UT, untransformed plants; L1–L3, transgenic potato plants expressing StnsLTP1 gene. [file Image_1.JPEG]
